# In Vivo Validation of the BIANCA Biophysical Model: Benchmarking against Rat Spinal Cord RBE Data

**DOI:** 10.3390/ijms21113973

**Published:** 2020-06-01

**Authors:** Mario P. Carante, Giulia Aricò, Alfredo Ferrari, Christian P. Karger, Wioletta Kozlowska, Andrea Mairani, Paola Sala, Francesca Ballarini

**Affiliations:** 1 INFN (Italian National Institute for Nuclear Physics), Sezione di Pavia, via Bassi 6, I-27100 Pavia, Italy; mario.carante@pv.infn.it; 2 CERN—European Organization for Nuclear Research, 1211 Geneva, Switzerland; giulia.arico@cern.ch (G.A.); alfredo.ferrari@cern.ch (A.F.); wioletta.kozlowska@cern.ch (W.K.); 3 Department of Medical Physics in Radiation Oncology, German Cancer Research Center (DKFZ), 69120 Heidelberg, Germany; c.karger@dkfz-heidelberg.de; 4 Medical University of Vienna, 1090 Vienna, Austria; 5 HIT (Heidelberg Ion-Beam Therapy Center), 69120 Heidelberg, Germany; andrea.mairani@med.uni-heidelberg.de; 6 INFN (Italian National Institute for Nuclear Physics), Sezione di Milano, via Celoria 16, I-20133 Milano, Italy; paola.sala@mi.infn.it; 7 Physics Department, University of Pavia, via Bassi 6, I-27100 Pavia, Italy

**Keywords:** head-and-neck tumors, ion beam radiotherapy, relative biological effectiveness (RBE), RBE models, carbon ions, protons, Monte Carlo simulations

## Abstract

(1) Background: Cancer ion therapy is constantly growing thanks to its increased precision and, for heavy ions, its increased biological effectiveness (RBE) with respect to conventional photon therapy. The complex dependence of RBE on many factors demands biophysical modeling. Up to now, only the Local Effect Model (LEM), the Microdosimetric Kinetic Model (MKM), and the “mixed-beam” model are used in clinics. (2) Methods: In this work, the BIANCA biophysical model, after extensive benchmarking in vitro, was applied to develop a database predicting cell survival for different ions, energies, and doses. Following interface with the FLUKA Monte Carlo transport code, for the first time, BIANCA was benchmarked against in vivo data obtained by C-ion or proton irradiation of the rat spinal cord. The latter is a well-established model for CNS (central nervous system) late effects, which, in turn, are the main dose-limiting factors for head-and-neck tumors. Furthermore, these data have been considered to validate the LEM version applied in clinics. (3) Results: Although further benchmarking is desirable, the agreement between simulations and data suggests that BIANCA can predict RBE for C-ion or proton treatment of head-and-neck tumors. In particular, the agreement with proton data may be relevant if the current assumption of a constant proton RBE of 1.1 is revised. (4) Conclusions: This work provides the basis for future benchmarking against patient data, as well as the development of other databases for specific tumor types and/or normal tissues.

## 1. Introduction

Cancer ion beam therapy is spreading more and more worldwide, with more than 220,000 patients treated and more than 100 facilities in operation by the end of 2019. While most patients have been irradiated with protons, about 28,000 have been treated with C-ions (www.ptcog.ch).

Due to the high dose localization in the Spread-Out Bragg Peak (SOBP) region, radiotherapy treatments with charged particles like protons or carbon ions are characterized by a highly conformal dose distribution in the tumor. Furthermore, heavy ions like carbon possess a higher biological effectiveness within the SOBP. This is thought to be due to a higher level of DNA damage clustering e.g., [1,2,3], which is less likely to be repaired. This is an advantage especially for patients with radioresistant tumors, like head-and-neck tumors [4].

As a consequence, the RBE variation along the beam needs to be known as precisely as possible. This is especially true if the dose is delivered by an active beam scanning technique as employed at CNAO in Pavia (Italy) and HIT in Heidelberg (Germany) because the RBE has to be predicted for each point in the treatment field.

The RBE is a complex quantity depending not only on radiation quality (that is, particle type and Linear Energy Transfer, or LET), but also on the considered effect level and thus fractional dose: in general, higher survival levels imply higher RBE values, and *vice versa*. In addition, more radioresistant cells, which in general have a lower α/β ratio, tend to show higher RBE values with respect to more radiosensitive cells, which are characterized by a higher α/β ratio.

Therefore, predicting RBE for heavy ions requires appropriate models. Currently, only three models are applied in clinics: the Local Effect Model (LEM) in Europe and Shanghai (China) e.g., [5,6], the Microdosimetric Kinetic Model (MKM) e.g., [7,8] and the “mixed-beam” model e.g., [9] in Japan. The LEM was first applied to tumors of the skull base, considering late reactions of the central nervous system (CNS) as the main endpoint; indeed, late effects in the CNS are the main dose-limiting factor for head-and-neck tumors. Since data on CNS radiation tolerance in patients are scarce, animal studies are necessary.

Although absolute dose values obtained in such studies may not be directly transferable to patient cases, relative values such as the RBE and its dependence on treatment parameters may be more applicable [10]. The rat spinal cord is known as a well-established model to investigate CNS late effects [11], and it has been applied to measure RBE values for C-ion therapy e.g., [10,12,13,14] and protons [15]. These are the main in vivo data that have been used to benchmark the clinically employed LEM version (LEM I), but also the more recent version LEM IV [6].

In the present work, the BIANCA (BIophysical ANalysis of Cell death and chromosome Aberrations) biophysical model, after extensive in vitro validation e.g., [16,17,18], was applied to establish a radiobiological database (i.e., alpha and beta cell survival parameters as a function of particle type and energy). The model was then tested against the available rat spinal cord data for carbon ion and proton irradiations [15]. Using the FLUKA Monte Carlo transport code [19,20,21,22], which is used at HIT in Heidelberg (Germany) and CNAO in Pavia (Italy) for treatment plan verification/re-calculation, the RBE is predicted for the experimental settings and compared to experimental data.

## 2. Results

### 2.1. RBE-LET Relationship

**Figure 1:**
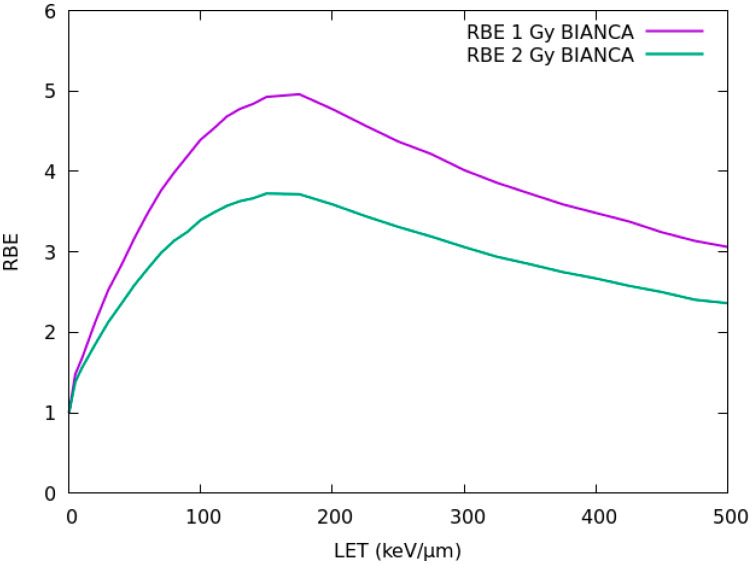
Relative biological effectiveness–Linear Energy Transfer (RBE-LET) relationship calculated by BIANCA for chordoma cell survival following irradiation with monochromatic carbon beams at 1 Gy (upper curve) or 2 Gy (lower curve) Carbon dose.

Figure 1 reports the RBE-LET relationship calculated by BIANCA for chordoma cell survival following irradiation with different monochromatic carbon beams at 1 Gy Carbon dose, which is of interest for the entrance channel in a typical therapeutic fraction, and 2 Gy Carbon dose, of interest for the SOBP. The calculations were performed as explained in Section 4.2. Although a monochromatic irradiation is different from the mixed-field scenario that is typically used in patients, these plots can be useful for interpreting the results that will be subsequently shown. Both at 1 Gy and 2 Gy, the RBE shows a steep increase up to a maximum LET, followed by a moderate decrease in the so-called “overkilling” region. As expected, the RBE at 1 Gy is higher than that at 2 Gy.

### 2.2. Carbon Ions: Dose Dependence

**Figure 2:**
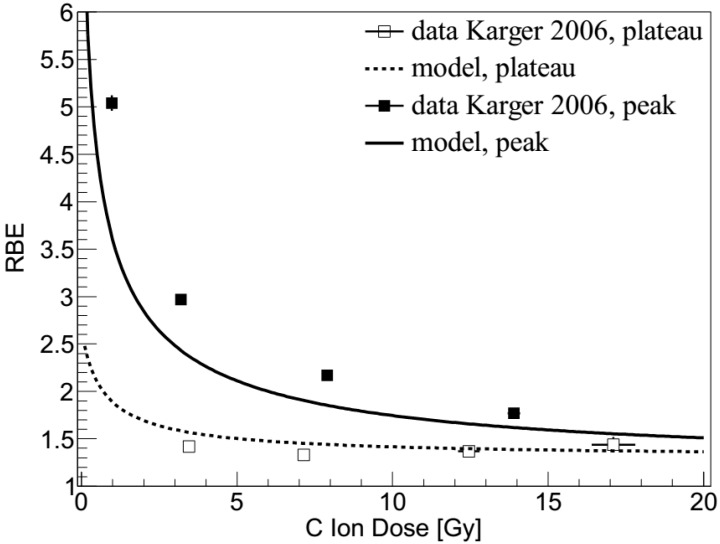
RBE as a function of carbon ion dose for irradiation of the rat spinal cord in the SOBP (upper line and filled data points) or in the entrance plateau (lower line and open data points). The lines are predictions by BIANCA, the points are experimental data taken from [10].

In Figure 2, RBE values calculated in the present work by BIANCA are compared with rat spinal cord experimental data [10,23]. In these experiments, the spinal cord of the animals was positioned either in the entrance plateau of a 270 MeV/u beam (dose-averaged LET: 13 keV/μm), or in the middle of a homogeneous 1-cm SOBP of a 140 MeV/u beam (dose-averaged LET: 125 ± 25 keV/μm). The animals were irradiated with 1, 2, 6, or 18 fractions, and RBE values with respect to 15 MV photons ranged from 1.33–1.44 (plateau) or 1.77–5.04 (peak). Further details on the experimental study can be found in [10,23], whereas the methods adopted to perform the calculations are described in Section 4.2 (RBE prediction by BIANCA for monochromatic beams) and 4.3 (interface between BIANCA and FLUKA to predict RBE for mixed fields).

### 2.3. Carbon Ions: LET Dependence

BIANCA calculations, performed as described in Section 4.2 and Section 4.3, were additionally compared to more recent data [12,13,14], where the spinal cord was located at six positions of a 6-cm SOBP (LET values: 16, 21, 36, 45, 66 and 99 keV/µm). The animals were irradiated either by a single fraction [12,14], or by two fractions [13]. RBE values relative to 15 MV photons were obtained.

**Figure 3:**
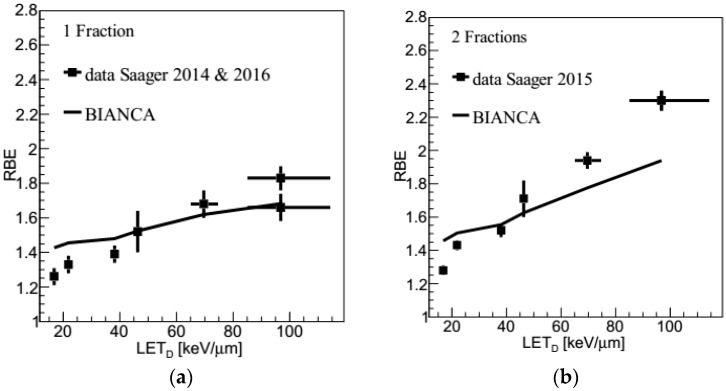
RBE as a function of dose-averaged LET for single-fraction (panel **a**) or two-fraction (panel **b**) irradiation of the rat spinal cord at different positions within the carbon-ion Spread-Out Bragg Peak (SOBP). The lines are predictions obtained by BIANCA, the points are experimental data taken from [12,14] (panel **a**), or [13] (panel **b**). In panel a, at 99 keV/µm the higher experimental value is from a repetition experiment [14] and is considered as more reliable.

In Figure 3a,b, the predictions of BIANCA are compared with these experimental data. Both for the single fraction and for the split dose (i.e., two-fraction) case, the predicted slope is smaller than for the experimental data. As a consequence, the RBE at lower LET is slightly overestimated, while at higher LET the RBE is underestimated, especially for split-doses.

### 2.4. Protons

**Figure 4:**
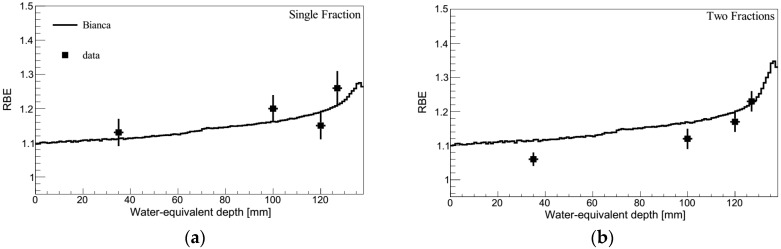
RBE as a function of depth for single-fraction (panel **a**) or two-fraction (panel **b**) proton irradiation of the rat spinal cord. The lines are predictions by BIANCA, the points are experimental data taken from [15].

Figure 4 shows predictions performed by BIANCA (following the methods described in Section 4.2 and Section 4.3) compared with RBE data for the rat spinal cord reported in [15], in the framework of the ongoing discussion whether the fixed RBE of 1.1 endorsed by ICRU [24] is still appropriate e.g., [25,26]. Analogous to the C-ion experiments, the spinal cord was positioned at four positions (35, 100, 120 and 127 mm, corresponding to LET values of 1.4, 2.7, 3.9 and 5.5 keV/μm) of a 6-cm proton SOBP ranging from 70–130 mm, and was irradiated either with single or split doses. Further details can be found in the original paper [15].

## 3. Discussion

Following interface with the FLUKA Monte Carlo transport code (as described in Section 4.3), for the first time the BIANCA biophysical model, described in Section 4.1, was benchmarked against in vivo data, obtained by C-ion or proton irradiation of the rat spinal cord. The C-ion data are those that have been used in the past to validate the LEM version currently used in clinics. More specifically, two C-ion experiments were considered: in the first one, the RBE dose-dependence was investigated by locating the animals in the entrance plateau or in the middle of a 1-cm SOBP, whereas in the second one, the LET-dependence was analyzed by irradiating the rat spinal cord at six positions of a 6-cm SOBP, with LET values in the range 16–99 keV/µm. In the LET experiment, the animals were irradiated either by a single fraction, or by two fractions.

Concerning the dose-dependence study, in the entrance plateau (Figure 2, lower line) the simulations show a very good agreement with the data, both qualitatively and quantitatively. This suggests that BIANCA may be applied for the evaluation of late side effects in the CNS. Concerning the SOBP (Figure 2, upper line), the simulations reproduce well the trend of the RBE dose-dependence. Quantitatively, however, there is a tendency to underestimate the data, although the degree of underestimation decreases with increasing dose. This suggests that BIANCA may underestimate the beam effectiveness in the distal SOBP region, where the LET is highest. It is worth mentioning that in the experimental study reported in [10] only the photon doses referred to measurements, whereas the carbon doses were predicted by the TPS. In a subsequent work [12], the authors found that the measured doses were lower than the TPS doses by a factor up to about 10%. This may imply that the discrepancy between simulations and data might be smaller than the one shown in Figure 2, since the data points reported in Figure 2 should be shifted towards lower doses. However, this is not automatic because the corresponding RBE values would increase as well. In addition to the experimental data, also LEM predictions are reported in [10]. According to the authors, in the peak, LEM shows a systematic underestimation of the experimental values by about 25%, whereas, in the plateau, LEM shows a (slight) RBE decrease with increasing the dose. As a consequence, LEM overestimates the point at the lowest dose (3.45 Gy) and underestimates that at the highest dose (17.1 Gy), both by about 20% [10].

In the LET-dependence study (Figure 3), the RBE at lower LET is slightly overestimated, while at higher LET the RBE is underestimated, especially for split-doses. The overestimation at the lower LET values may seem in contrast with the results of the dose-dependence investigation, where BIANCA shows a very good agreement with the data at low LET. However, it has to be taken into account that a comparison between these two scenarios is problematic as the two beam configurations result in different RBE predictions, which are then compared with data of two independent experiments. Furthermore, the two sets of experimental data use two different dose ranges (i.e., about 15–27 Gy for the LET study, 1–17 Gy for the dose study), and the LET validation is done only for relatively high doses. This suggests that the overestimation shown by BIANCA at the lower LET values might be only related to these very high doses, whereas at therapeutic doses, like those considered in the dose-dependence study, BIANCA agrees very well with the data. Concerning the higher LET values, this work shows that at high doses, like those used in the single fraction experiment (Figure 3a), BIANCA agrees well with the data, whereas at lower (fractional) doses, down to therapeutic values like those reported in the left region of Figure 2, BIANCA tends to underestimate the beam effectiveness, although the underestimation factor might be smaller than that shown by Figure 2, as already mentioned above. In the future, we will investigate whether this underestimation at high LET also holds for patient cases.

Concerning possible comparisons with LEM, while BIANCA tends to overestimate the data at low LET and to underestimate them at high LET, the LEM clinical version, also called LEM I, underestimates the data for all considered LET values; comparisons between LEM I and these data can be found in [13]. However, from a quantitative point of view, the relative discrepancy between calculations and data tends to be lower for BIANCA than for LEM I. More specifically, BIANCA shows a higher discrepancy with the data only at the lowest LET (16 keV/µm), where the discrepancy is about 13% (single fraction) or 14% (two fractions), whereas the LEM I discrepancy is 9% (1 fx) or 5% (2 fx). On the contrary, for all other five LET values the discrepancy found in this work with BIANCA is lower than that reported in [13] for LEM I. For instance, at mid-SOBP (45 keV/µm) the BIANCA discrepancy is 0% (1 fx) or 4% (2 fx), whereas that of LEM I is 19% (1 fx) or 18% (2 fx); in the distal end (99 keV/µm), these numbers become 8% (1 fx) or 15% (2 fx) for BIANCA, to be compared with 29% (1 fx) or 28% (2 fx) for LEM I. In [13], comparisons between the experimental data and the subsequent LEM IV version are also reported. Although at the moment LEM IV is not used in C-ion clinical practice, it may be worth mentioning that only at 99 keV/µm (both 1 fx and 2 fx) and 66 keV/µm (2 fx) LEM IV shows a smaller discrepancy with the data with respect to BIANCA, whereas for all other cases the reverse holds.

The C-ion study was then extended to protons, comparing BIANCA simulations with RBE data for the rat spinal cord obtained by locating the animals at four different depth positions of a 6-cm proton SOBP, and performing the irradiations either by a single or by a split dose [15]. Qualitatively, the simulations reproduced well the RBE increase with LET, and thus, with depth. The quantitative agreement with the single fraction data was also very good, since the simulation outcomes were always within the experimental error bars. While the simulation of the split-dose experiment revealed similar results as the single-fraction study, the experimental data tend to be lower than that of the single-fraction data, and thus also lower than the split-dose simulations. However, according to the authors, the split-dose experimental results should rather be regarded as a confirmation of the single-dose study. While one would expect that the split-dose RBE is not lower than the single-fraction RBE, the difference (up to 5–7%) is in the limit of the biological experimental accuracy [15]. Overall, the comparison with the proton data suggests that BIANCA may be used to predict late effects in the CNS not only for C-ions but also for protons. This may be important if it is decided that the current approach based on a constant RBE of 1.1 needs to be revised in the future.

## 4. Materials and Methods

### 4.1. The BIANCA Model: Assumptions, Parameters and Structure

The radiobiological simulations performed in this work were based on the BIANCA biophysical model, which predicts chromosome damage and cell death by different radiation types. BIANCA, which is implemented as a Monte Carlo simulation code, is based on the following main assumptions: i) ionizing radiation can induce “critical lesions” (CLs) of the DNA, where a CL is defined as a lesion that breaks the chromatin giving rise to two independent chromosome fragments; ii) distance-dependent mis-rejoining of such fragments, or fragment un-rejoining, produces chromosomal aberrations; iii) certain aberration categories (that is dicentrics, rings, and large deletions, where “large” means visible when chromatin is condensed) lead to clonogenic cell death.

Since these assumptions have been discussed in detail in previous works e.g., [27,28], only a few key issues will be addressed here. In particular, the CL yield (i.e., mean number of critical lesions per unit dose and per unit DNA mass) is an adjustable parameter of the model, because the features of the critical DNA lesions leading to chromosomal aberrations and cell death are still an open question. The value of this parameter is strongly influenced by radiation quality (that is particle type and energy), although it also depends on the characteristics of the target cell. In general, the yield of CLs yield increases with LET (excluding very high LET values, where an overkill effect may occur) as well as with the cell radiosensitivity. As in our previous work, the dependence of the rejoining probability on the (initial) fragment-end distance was modeled by a step function; the threshold distance was fixed to the average distance between the centers of two adjacent chromosome territories. Although according to recent works an exponentially-decreasing function may be better for modeling of some specific intra-chromosome aberration types [29,30], a step function has been shown to be adequate to model the main aspects of cell death [16,17,18,27,31,32]. As in previous works, we assumed that each chromosome fragment has a certain probability, *f,* to remain un-rejoined, even when possible “partners” are available within the threshold distance. In BIANCA, *f* is the second, and last, adjustable parameter. The assumed link between chromosomal aberrations and cell death arises from experimental studies showing a one-to-one relationship between the logarithm of the cell surviving fraction and the average number of dicentrics, rings, and deletions per cell that were scored in metaphase e.g., [33,34].

As described previously e.g., [16,27,35], the main input information for running a simulation with BIANCA consist of the radiation type (photons, light ions or heavy ions), the yield of CLs and the *f* value, the shape of the cell nucleus (that can be either spherical or cylindrical) and its size, as well as the treatment parameters LET and absorbed dose. While for photon irradiation the CLs induced at a given dose are uniformly distributed within the cell nucleus, for light ions like protons and α-particles they are placed along (parallel) straight lines representing the various primary particles traversing the nucleus. In case of irradiation by heavy ions like Carbon, each CL induced by a given primary ion has a 0.5 probability to be placed along the primary-ion path, and a 0.5 probability to be created at a certain radial distance with respect to the primary ion path, to take into account the effects of high-energy secondary electrons, or “delta rays”. This 0.5 value derives from the consideration that 50% of the total energy deposition derives by interactions mainly occurring within the particle track core, whereas the remaining 50% derives from ionizations induced by secondary electrons, mainly occurring in the “penumbra” [36]. Afterwards, BIANCA identifies the chromosomes and the chromosome-arms that have been hit by each CL, simulates the end-joining process of the various chromosome fragments, and reproduces the scoring of dicentrics, rings, and deletions that are visible in metaphase (also called “lethal aberrations”). A cell without any lethal aberration is scored as a surviving cell, otherwise it is counted as a dead cell. For each considered dose level, the process is repeated until one obtains the desired statistical significance. The repetition for different dose levels allows for simulating a dose-response curve for lethal aberrations, and thus for cell survival. Further details on the irradiation procedure can be found in [18], whereas a detailed description of the simulation of interphase chromosome territories and arm domains is reported in [29,30].

### 4.2. Construction of a Radiobiological Database and RBE Prediction

As mentioned in Section 4.1, in BIANCA the CL yield depends not only on radiation quality but also on the target cell characteristics. Therefore, in principle, the CL yield for a given radiation quality should be separately adjusted for each cell line. However, we have recently developed an approach to predict the ion-survival of the cell line of interest starting from the ion-survival of a reference cell line, as well as the photon response of both lines [16]. According to this approach, the CL yield (mean number of CLs per unit length of the primary-ion path, expressed as CL/μm) to be used as an input to predict the survival of the cell line of interest for a given radiation quality, can be derived as follows:

(1) $$\frac{CL}{\mu m}={\left(\frac{CL}{\mu m}\right)}_{ref}\cdot \left[\left(\frac{CL}{Gy\cdot cell}\right)/{\left(\frac{CL}{Gy\cdot cell}\right)}_{ref}\right]\cdot \frac{V_{ref}}{V}$$

In Equation (1), (CL/μm)_ref_ is the CL yield used for the reference cell line irradiated with the same radiation quality, whereas (CL⋅Gy^−1^⋅cell^−1^) and (CL⋅Gy^−1^⋅cell^−1^)_ref_ are the CL yields (mean number of CLs per unit dose and per cell) used to simulate photon irradiation of the cell line of interest and the reference cell line, respectively. Finally, V_ref_ and V represent the nucleus volume of the reference cell line and the cell line of interest, respectively. In a previous study, V79 cells have been chosen as a reference, and V79 cell survival curves have been simulated for a wide range of particle types and LET values. Afterwards, the aforementioned formula has been successfully applied to predict the survival of CHO cells irradiated by two opposing fields of C-ions or protons at HIT [18].

In the present work, we used the approach described above to derive a radiobiological database for chordoma patients, both for the tumor and for the adjacent normal tissue. Like in previous works, the cell nucleus was modeled by a cylinder with 6-µm height and 6-µm radius. Since chordoma is a rare tumor and basically no radiobiological data were available when LEM was introduced clinically, the LEM authors decided to use α_X_ = 0.10 Gy^−1^ and β_X_ = 0.05 Gy^−2^, leading to a reasonable α_X_/β_X_ ratio of 2 Gy. Nowadays, additional data are available, both for in vitro [37,38] and for in vivo [39] settings. In particular, Henderson and co-workers irradiated chordoma patients (with a tumor volume of about 100 cm^3^) with a total dose D = 35 Gy and a fractional dose d = 7 Gy getting a TCP (Tumor Control Probability) of about 60%, and they estimated an α_X_/β_X_ ratio of 2.45 Gy:

TCP = exp[−N⋅exp(−α_X_D − β_X_Dd)] (2)

N was calculated assuming a cell density of 10^7^ cells/cm^3^, as reported in the literature [40,41]. From this calculation, we obtained α_X_ = 0.159 and β_X_ = 0.065, which appears to be a reasonable estimation for photon irradiation of chordoma in vivo. Importantly, the α_X_/β_X_ ratio of 2.45 Gy is still consistent with the value α_X_/β_X_=2 Gy adopted in clinical LEM applications to minimize normal tissue damage.

Afterwards, we adjusted the CL yield to reproduce this photon survival curve by our simulations, and we included this (photon) CL yield in Equation (1) to obtain ion CL yields. This allowed us to derive the CL yields to predict cell survival for many different particle types and energies. Finally, fitting of each of these survival curves by the Linear-Quadratic model S(D) = exp(-αD-βD^2^) was performed to produce a radiobiological database, i.e., a table of alpha and beta coefficients as a function of particle type and energy.

The corresponding RBE values were calculated based on the LQ-model as follows e.g., [42]:

(3) $$RBE=2\beta_{i}\left[-\alpha_{X}+\sqrt{\alpha_{X}^{2}-4\beta_{X}\ln S}\right]/2\beta_{X}\left[-\alpha_{i}+\sqrt{\alpha_{i}^{2}-4\beta_{i}\ln S}\right]$$

In Equation (3), α_X_ and β_X_ are the photon coefficients, α_i_ and β_i_ are the ion coefficients (at a given ion energy, and thus LET), and S is the considered survival level.

### 4.3. Interface between BIANCA and the FLUKA Monte Carlo Transport Code

Analogous to our previous work on in vitro irradiation of CHO cells by two opposing beams [18], the radiobiological table obtained as described in Section 4.2 was used by the FLUKA Monte Carlo transport code (version 2020.0, www.fluka.org).

More specifically, FLUKA calculated the necessary information (that is particle type and energy, and absorbed dose) based on a voxel-by-voxel approach: when a certain amount of energy was absorbed in a voxel due to a given particle type of given energy (and thus LET), FLUKA used the corresponding α_i_ and β_i_ values from the radiobiological table and calculated the respective values for the mixed beam conditions based on the TDRA (Theory of Dual Radiation Action) [43] as described in [42], i.e.,

(4) $$\alpha =\frac{\sum_{i=1}^{n}\alpha_{i}D_{i}}{\sum_{i=1}^{n}D_{i}}$$

(5) $$\sqrt{\beta }=\frac{\sum_{i=1}^{n}\sqrt{\beta_{i}}D_{i}}{\sum_{i=1}^{n}D_{i}}$$

Here, D_i_ is the absorbed dose (in the considered voxel) due to the i-th particle calculated by FLUKA, α_i_ and β_i_ are the corresponding radiobiological parameters provided by BIANCA, and α and β are their values under mixed-beam conditions. The corresponding cell survival fraction, RBE-weighted dose (D_RBE_) and RBE were then calculated as follows:

(6) $$-\ln S=\alpha D+\beta D^{2}$$

(7) $$D_{RBE}=\left[\sqrt{\alpha_{X}^{2}-4\beta_{X}\ln S}\right]/2\beta_{X}$$

(8) $$RBE=\frac{D_{RBE}}{D}$$

In Equations (7) and (8), α_X_ and β_X_ are the photon parameters provided by BIANCA.

## 5. Conclusions

Based on the BIANCA biophysical model, a radiobiological database was constructed to predict RBE values for head-and-neck tumor irradiation with C-ions or protons. Establishing an interface with the FLUKA Monte Carlo transport code, this allowed benchmarking BIANCA against in vivo data for the first time. The RBE values predicted by BIANCA were in line with experimental RBE values obtained by irradiating the rat spinal cord at different depth positions along C-ion or proton SOBPs. Although further benchmarking is also necessary to investigate whether this approach can be extended to patient cases, this work suggests that BIANCA may be applied to predict RBE values of normal CNS tissue response in carbon ion or proton treatments. The agreement with the proton data may be especially relevant if the current assumption of a constant proton RBE of 1.1 is revised in the future. Further experimental in vivo data at lower fractional doses, comparable to those used for patient treatment, are needed.
